# Functionalization of the NiTi Shape Memory Alloy Surface by HAp/SiO_2_/Ag Hybrid Coatings Formed on SiO_2_-TiO_2_ Glass Interlayer

**DOI:** 10.3390/ma13071648

**Published:** 2020-04-02

**Authors:** Karolina Dudek, Mateusz Dulski, Bożena Łosiewicz

**Affiliations:** 1 Refractory Materials Division in Gliwice, ŁUKASIEWICZ Research Network—Institute of Ceramics and Building Materials, Toszecka 99, 44-100 Gliwice, Poland; 2 Institute of Materials Engineering, University of Silesia in Katowice, 75 Pulku Piechoty 1A, 41-500 Chorzow, Poland; mateusz.dulski@smcebi.edu.pl (M.D.); bozena.losiewicz@us.edu.pl (B.Ł.); 3 Silesian Center for Education and Interdisciplinary Research, University of Silesia in Katowice, 75 Pulku Piechoty 1A, 41-500 Chorzow, Poland

**Keywords:** hybrid coatings, SiO_2_-TiO_2_ glass, SiO_2_/Ag nanocomposite, surface modification, hydroxyapatite (HAp), NiTi shape memory alloy (SMA), pitting

## Abstract

The surface modification of NiTi shape memory alloys is a method for increasing their multi-functionalities. In our solution, hydroxyapatite powder was mixed with a chemically synthesized silicon dioxide/silver (nSiO_2_/Ag) nanocomposite in a different weight ratio between components (1:1, 5:1, and 10:1) and then electrophoretically deposited on the surface of the NiTi alloy, under various time and voltage conditions. Subsequently, uniform layers were subjected to heat treatment at 700 °C for 2 h in an argon atmosphere to improve the strength of their adhesion to the NiTi substrate. A change in linear dimensions of the co-deposited materials during the sintering process was also analyzed. After the heat treatment, XRD, Raman, and Scanning Electron Microscopy (SEM) + Energy Dispersive Spectrometer (EDS) studies revealed the formation of completely new composite coatings, which consisted of rutile and TiO_2_-SiO_2_ glass with silver oxide and HAp particles that were embedded into such coatings. It was found that spalling characterized the 1:1 ratio coating, while the others were crack-free, well-adhered, and capable of deformation to 3.5%. Coatings with a higher concentration of nanocomposite were rougher. Electrochemical impedance spectroscopy (EIS) tests in Ringer’s solution revealed the capacitive behavior of the material with high corrosion resistance. The kinetics and susceptibility to pitting corrosion was the highest for the NiTi electrode that was coated with a 5:1 ratio HAp/nSiO_2_/Ag hybrid coating.

## 1. Introduction

NiTi shape memory alloys (SMAs), with a chemical composition that is similar to the equilibrium, have outstanding properties, such as one-way-, two-way-shape memory effect (SME), superelasticity, and acceptable biocompatibility. They are the most common SMAs applied in a wide range of biomedical fields due to these features—for implants, especially in orthopedics, as well as surgical tools in medicine [1,2,3,4]. Unfortunately, the long-term use of NiTi alloy implants necessitates the improvement of their corrosion resistance and biocompatibility [5,6,7]. This goal might be achieved by modifying the alloy surface by protective coatings made of ceramics [8,9,10,11], polymers [12], metals [13], diamond-like carbon—DLC [14], or complex composite layers [15,16,17,18].

It is well known that the most optimal binding properties are linked to calcium phosphates (CaPs) with a chemical composition that is similar to that of the human bone [19,20,21]. Among the various forms of CaPs ceramics, most attention is focused on hydroxyapatite (HAp), β-tricalcium phosphate (β-TCP), and biphasic calcium phosphates (BCP) [22,23,24], as these types of ceramics belong to the group of biomaterials characterized by high biocompatibility with bone tissue (which improves bone regeneration), high compressive strength, hardness (similar to tissue), and biotolerance as well as good corrosion resistance, ensuring cell proliferation, bioactivity, etc. Moreover, apatite materials display relatively high biocompatibility with both hard and soft tissues. They are commonly applied in dentistry, maxillo-facial surgery, and orthopedics, as drug carriers, a bioactive scaffold allowing for the growth of bone tissue, and a good bonding with bone tissue stem, contributing to the improvement of interlayer adhesion. Unfortunately, other mechanical properties of calcium phosphate ceramics, such as friability (low fracture toughness), low formability in contact with tissues, low tensile strength, or low Weibull modulus, cause that its application is limited. Moreover, pure apatite might be prone to form a microbial biofilm on its surface, which favors the development of various infections [19,20,21,22,23,24]. Calcium phosphates-based ceramic is frequently used to produce coatings on metallic implants, including NiTi alloys, by various surface modification methods [10,11,25,26,27,28]. 

An alternative, recently developed approach is the formation of hybrid composite layers combining the properties of different materials e.g., nanosilver-doped CaPs layers [29,30,31,32], which can improve the alloy biocompatibility and exhibit antibacterial properties. However, the silver content in such coatings must be relatively low (<1 wt.%), because elevated concentrations have resulted in high toxicity not only for bacteria, but also for human body cells [33,34]. Moreover, a concentration of silver lower than 1 wt.% in the Ag-hydroxyapatite coating that was deposited on a titanium substrate or in Ag/SiO_2_-β-TCP coatings stimulates cell proliferation without the cytotoxicity effect [33,35]. Another idea that is aimed at enhancing the functionality of coatings is to use a combination of calcium phosphates with pure silica or silica doped by low concentrations of metal in the form of ions or nanoparticles (e.g., silver, copper). Silica-based systems may be useful in the formation of bone structures—they improve the regeneration process (e.g., after fractures), accelerate calcification, and support the treatment of bone defects in the hip, knee, and spine [36]. Furthermore, silica glass and silica-based composites are becoming increasingly useful in orthopedics and other medicine branches [37,38].

In the case of surface modification methods dedicated to NiTi SMA, the low-temperature ones are crucial. The NiTi alloy is very sensitive to temperature increase, which can lead to the decomposition of the B2 parent phase to the equilibrium (Ti_2_Ni and/or Ni_3_Ti) or non-equilibrium phase (such as Ni_4_Ti_3_). High temperatures and long heat treatment times lead to the precipitation of Ni_3_Ti particles, while, in the case of lower temperatures or shorter times, the decomposition process occurs through metastable intermediate phases. The Ni_4_Ti_3_ phase is formed during low-temperature aging in the B2 phase. Extending the aging time results in the formation of the Ni_3_Ti_2_ phase with a simultaneous dissolution of the Ni_4_Ti_3_ particles, followed by the precipitation of the equilibrium Ni_3_Ti phase particles. Consequently, the decomposition of the B2 parent phase influences the shape memory and the superelasticity effect [1,10,11,39,40]. One of the interesting methods for modifying the surface seems to be electrophoretic deposition (EPD) [41,42,43,44,45]. Electrophoresis is particularly recommended for the formation of coatings on substrates with complicated shapes and morphology, such as implants [43]. EPD also provides the possibility to control coating thickness, which is extremely important in the case of alloys with shape memory effects. It turned out that SME might be limited or completely blocked by too thick and/or too rigid coatings. It forces surface modification while using thin layers up to a few micrometers in thickness. 

Another significant problem that is connected with the layers formed on shape memory alloys is the deformation of the coat-forming material. Unfortunately, relatively scarce information regarding this type of studies on NiTi alloys, especially the ones with a surface that is functionalized by hybrid coatings, makes it impossible to predict the behavior of such materials when they are subjected to deformation. However, it is known that pure ceramic systems have lower tensile than compressive strength. Compressive and tensile stresses in the case of an implant functionalized by ceramics are different in their various parts, which can lead to the cracking or delamination of layers. On the other hand, there are no systematic studies on the impact of deformation on hybrid coatings, especially those that are composed of calcium phosphate and silica, or more complex system.

Multifunctional coatings produced on alloys used in medicine should be designed to accelerate the osseointegration process and reduce the risk of inflammation and the release of harmful compounds from the implant, i.e., corrosion products into the body. According to the latest trends in implantology, this is achieved by covering the biomaterial surface with coatings having a chemical composition similar to that of the surrounding tissues. Bioactive coatings support the regeneration process by stimulating the reconstruction of tissues that surround the implant; they can be a source of tissue-forming elements and a carrier of medicinal substances, but, above all, they must meet the strict requirements for corrosion resistance in a biological environment [46,47,48].

The present work summarizes preliminary studies that present a new way of improving the biocompatibility and functionalizing the surface of NiTi shape memory alloys. First, a high corrosion resistance was obtained by functionalizing the surface with a biocompatible thin TiO_2_ film through passivation in an autoclave. Next, the new hybrid coating that consists of hydroxyapatite (HAp) and nanocomposite silica-silver (nSiO_2_/Ag) was deposited on the passivated surface by the EPD method. The paper summarizes the results that were obtained in studies on the morphology, topography, structure, as well as the deformation ability of the multifunctional layers formed on the NiTi shape memory alloy. Special focus was placed on determining the in vitro corrosion resistance of the developed HAp/nSiO_2_/Ag hybrid coatings in Ringer’s solution, including the mechanism and kinetics of pitting corrosion.

## 2. Materials and Methods

### 2.1. Substrate Treatment Procedure

A commercially available NiTi alloy in β-phase (B2) with characteristic temperatures of martensitic transformations that were below ambient temperature was used as a substrate for hybrid coatings deposition. The samples were polished by SiC papers up to 2000-grid, diamond suspension down to 1 μm and, finally, a 0.1-μm colloidal silica suspension. Before deposition, the substrate was passivated in the air autoclave at 134 °C for 30 min. to form a thin amorphous TiO_2_ layer [25].

### 2.2. Suspension Preparation and Formation of Coatings

The coatings were deposited using the EPD technique from a colloidal suspension having a concentration of 0.1 wt.% HAp powder (Sigma Aldrich, Saint Louis, MI, USA) and nSiO_2_/Ag nanocomposite powder in 96% ethanol (Avantor Performance Materials, Gliwice, Poland). The silver-silica nanosystem was prepared according to the procedure that was described by Peszke et al. [49]. The mixtures of 10:1, 5:1, and 1:1 ratio of HAp and nanocomposite were used to prepare a colloidal suspension. Before deposition, the suspensions were placed in the ultrasonic bath for 2 h. A NiTi alloy was used as a cathode, and platinum as a counter electrode. Cataphoretic deposition was performed under a wide range of voltage, reaching from 5 to 50 V and time periods from 0.5 to 5 min. Next, the green coatings were dried at room temperature for 24 h. Subsequently, the uniform layers were subjected to heat treatment at 700 °C under an argon atmosphere for 2 h to sinter the ceramics particles and increase the coating’s adhesion to the metallic NiTi substrate.

### 2.3. Coating Characterization

The structures of the material and phase identification were investigated by an X’PertPro MPD PANalytical X-ray diffractometer (Malvern PANalytical, Almelo, The Netherlands) with CuKα radiation. The coated NiTi alloy was examined by the grazing incidence X-ray diffraction technique (GIXD). The GIXD patterns were measured at a constant incidence angle of 1.2° at room temperature. The qualitative analysis was performed with HighScore Plus software (version 4.6, Malvern Panalytical, Almelo, The Netherlands) while using the ICDD PDF 4+ database.

A WITec confocal CRM alpha 300 R spectrometer (WITec Company, Ulm, Germany) that was equipped with a laser operating at 532 nm with approximately 50 mW radiation power and a high sensitivity back-illuminated Newton-CCD camera (Andor Technology, an Oxford Instruments Technology, Belfast, Northern Ireland) were applied to identify structural data. The Raman spectra were collected with an integration time of 20 s with 20 accumulations. The data were collected at room temperature with a 100×/0.9 NA Olympus lens (Olympus Corporation, Shinjuku, Tokyo, Japan), at a nominal resolution of 3 cm^−1^ in the 120–4000 cm^−1^ range. The data were subjected to the procedure of cosmic ray removal and baseline correction, whereas the band fitting analysis was performed using a Lorentz–Gauss function with a minimum number of components.

Scanning electron microscopy (SEM) data were obtained by means of TESCAN Mira 3 LMU (TESCAN, Brno, Czech Republic) that was equipped with an Energy Dispersive Spectrometer (EDS) (Oxford Instruments, Abingdon, UK), which enabled determining the microstructure and performing a chemical analysis. Images were collected by secondary electrons (SE) and backscattered electrons (BSE). The measurements were carried out on samples that were covered by a carbon layer, using Quorum Q150T ES equipment (Quorum Technologies, East Sussex, UK). The deformation ability was tested with an external device, whereas the surface of deformed coatings was thoroughly investigated under a scanning electron microscope. 

A Leica DCM8 optical profilometer (Leica Microsystems, Wetzlar, Germany) was applied for three-dimensional (3D) surface analysis of the topography. The roughness index Sa (average roughness) was estimated from the area of ca 1.3 × 1.6 mm.

A TMA-92 Dilatometer (Setaram Instrumentation, Caluire, France) was used to measure the changes in linear dimensions of the initial powders (HAp, nSiO_2_/Ag nanocomposite) versus temperature. The studies were carried out at temperatures that ranged from 20 to 1300 °C, with a heating rate of 20 °C/min. in an argon atmosphere.

The adhesive strength was tested according to ASTM-F1044 standard. A sample with a modified surface was stuck to unmodified one with epoxy resin-based adhesive and then subjected to the effect of shear forces. The adhesive strength test was carried out with a universal testing machine using a 10 kN load cell and a crosshead speed of 1.0 mm/min. The adhesion strength was calculated from the fracture force.

The pitting corrosion resistance of the NiTi alloy before and after surface modification was tested in Ringer’s solution with a pH of 7.4 ± 0.1 (Table 1). A 4% NaOH solution and 1% C_3_H_6_O_3_ solution were used to adjust the pH of the Ringer’s solution. Ultra-pure water (Millipore) with a resistivity of 18.2 MΩ cm was used to prepare all of the solutions. The sample corrosion behavior tests were carried out under thermostated conditions at 37.0 ± 0.1 °C. Immediately before starting the measurements, a fresh portion of the solution was deaerated in 99.999% argon for 30 min.

In vitro corrosion tests were carried out in a classic three-electrode system. During electrochemical measurements, the tested sample was a working electrode (WE), the counter electrode was a platinum grid (CE), and the reference electrode (RE) was used in the form of a saturated calomel electrode (SCE). The RE was inserted in the measuring system through the Luggin capillary. The one-sided geometric surface of the sample for electrochemical tests was 1.5 cm^2^. The other side of the WE was insulated with a chemical resistant adhesive. The WEs were rinsed and then washed twice for 30 min. in an ultrasonic bath using ultrapure water and, next, immediately placed in the electrolyser. The tested electrodes were placed vertically in a 250 cm^3^ electrolytic cell. Four types of samples were tested: S1—NiTi alloy after mechanical polishing, S2—NiTi alloy after passivation in a steam autoclave, S3—NiTi alloy after sterilization with the HAp/nSiO_2_/Ag coating (10:1, 40 V/90 s) sintered at 700 °C for 2 h in argon, and S4—NiTi alloy after sterilization with the HAp/nSiO_2_/Ag coating (5:1, 50 V/120 s) that was sintered at 700 °C for 2 h in argon (see Section 2.2). During the tests, a stream of argon was kept above the surface of the solution to ensure an inert atmosphere in the measuring cell.

The computer-controlled Autolab/PGSTAT30 ECO CHEMIE electrochemical system (Eco Chemie BV, Utrecht, The Netherlands) was used for the corrosion tests. The open circuit potential (*E*_OC_) was registered for 120 min. of the tested electrode exposure to a corrosive environment. The *E*_OC_ values were treated as approximate values of the corrosion potential (*E*_cor_).

Electrochemical impedance spectroscopy (EIS) measurements were potentially carried out at a stabilized *E*_OC_ value in the frequency range from 50 kHz to 2 mHz. Ten frequencies per current decade and the excitation signal in the form of a sine-wave with an amplitude of 10 mV were used. The impedance data were analyzed with electrical equivalent circuits while using the EQUIVCRT program (Eco Chemie BV, Utrecht, The Netherlands) and approximation by the complex non-linear least squares (CNLS) method. The electrical equivalent circuits were defined according to the circuit description code that was proposed by Boukamp [50]. 

The recording of anode polarization curves began with a potential 150 mV, which was more negative in relation to the stabilized *E*_OC_ value, and then continued with the polarization rate *v* = 1 mV s^−1^ towards the anode potentials. Next, the cathode-anode transition was recorded up to the breakdown potential (*E*_b_), at which point pitting appeared. After reaching the specified potential value, the polarization direction was reversed and the measurement proceeded towards the cathodic potentials up to the protection potential (*E*_p_). This way, a return curve was obtained, which did not coincide with the original curve over a certain section. The polarization curve formed a hysteresis loop, whose width determined the value of the tested material’s susceptibility to pitting corrosion. The resulting curve *j = f(E)* was later presented in a semi-logarithmic system, which allowed for the analysis of key current and potential values on the polarization curve.

## 3. Results and Discussion

### 3.1. Microstructure and Structural Investigations of the Deposited Coatings

**Figure 1:**
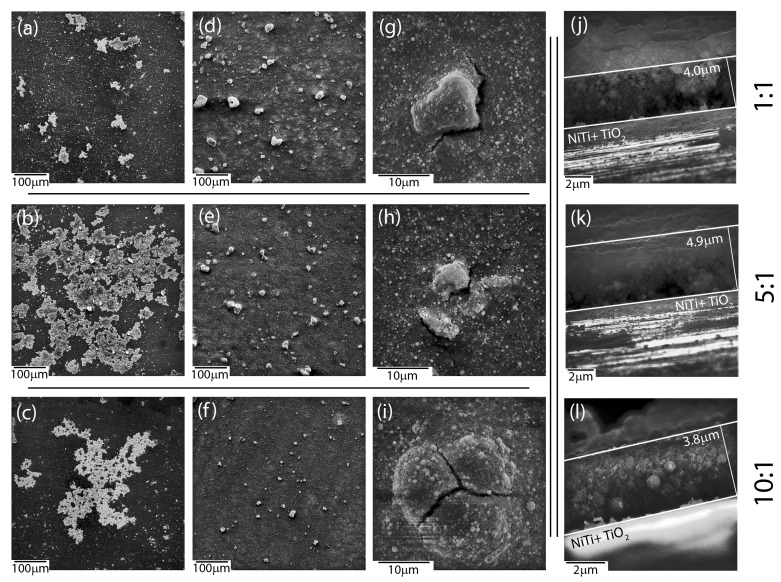
Scanning electron microscopy (SEM) images of coatings deposited under different conditions with different HAp: nSiO_2_/Ag ratios: 1:1 20 V/60 s (**a**), 5:1 20 V/60 s (**b**), 10:1 20 V/60 s (**c**), 1:1 50 V/240 s (**d**), 5:1 50 V/120 s (**e**), 10:1 40 V/90 s (**f**), 1:1 60 V/120 s (**g**), 5:1 60 V/120 s (**h**), 10:1 60 V/120 s (**i**). Cross-sectional images of samples: 1:1 50 V/240 s (**j**), 5:1 50 V/120 s (**k**) and 10:1 40 V/90 s (**l**).

Microscopic observations revealed an influence of the applied deposition parameters on the layers’ quality and enabled selecting optimal conditions for the production of layers with the most suitable material homogeneity. Coat-forming materials were subjected to agglomeration in larger or smaller objects, regardless of the mutual ratio between ceramic materials accessible in a colloidal suspension, according to SEM investigations. Moreover, the morphology of newly formed layers was strongly affected by the deposition conditions and mutual weight ratio between HAp and nSiO_2_/Ag (Figure 1). As a result, hybrid coatings that formed at low voltage (5–30 V) and/or short times (30–90 s) turned to be heterogeneous in terms of the distribution of the ceramic material on the NiTi substrate (Figure 1a–c). A decrease of hydroxyapatite weights in a colloidal suspension ensured the formation of ever-larger objects with irregular shapes. However, coatings that were prepared at those conditions at higher magnifications were crack-free and continuous in the vicinity of the coat-forming material. Another situation was observed in the case of coatings prepared at higher voltage values (60 V) and a longer deposition time (120 s). Here, the coat-forming material formed continuous layers with heterogeneously distributed agglomerates of irregular shape and variable size. Nevertheless, these layers were affected by cracks that were observed in the vicinity of the aggregates, which might influence coatings’ delaminating as well as provide a way of ion migration from the substrate (Figure 1g–i). Thus, continuous and crack-free coatings were formed at intermediate voltage values (40–50 V). However, an increase of hydroxyapatite concentration in relation to the concentration of silica-silver nanocomposite influenced the formation of coatings, which become more continuous and homogenous in terms of material distribution. Finally, the use of 50 V and 240 s for 1:1, 50 V and 120 s for 5:1, and 40 V and 90 s for 10:1 resulted in the formation of a relatively homogeneous coatings on the entire surface of the passivated alloy (Figure 1d–f) having a thickness of approximately 4–5 μm (Figure 1j–l). These data clearly shows that the increase of hydroxyapatite content in the colloidal suspension was correlated with a shorter time being needed to perform coatings of similar thickness. The estimation of the thickness and most optimal degree of functionalities is extremely crucial for the functionalization of shape memory alloys.

**Figure 2:**
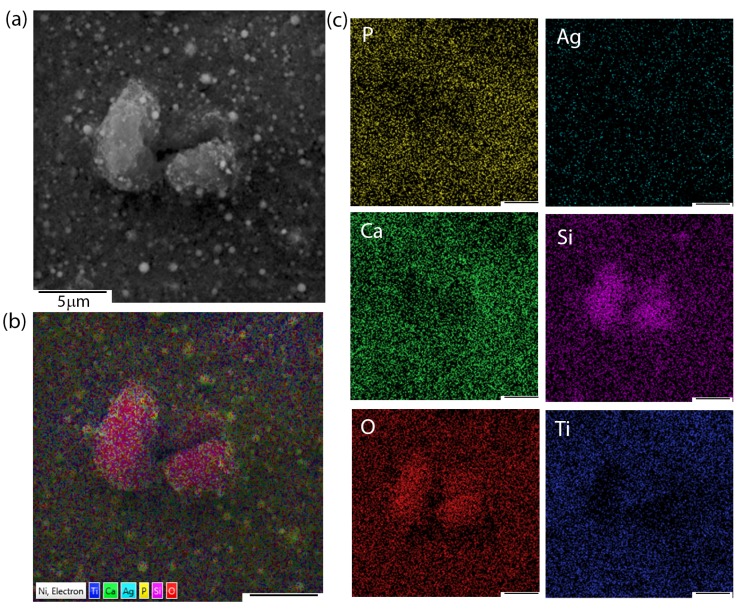
SEM image of 5:1 coating (**a**), multi-layer elemental distribution (**b**) and chemical composition imaging of elements (**c**).

Apart from microstructural observations, SEM + EDS imaging and chemical distribution analysis of individual elements were performed. Figure 2 presents the exemplary chemical images of coatings composed of HAp and nSiO_2_/Ag in 5:1 weight ratio and at 50 V/240 s. Interestingly, the nature of aggregates that were observed during SEM microstructure analysis resulted from a higher concentration of silicon and oxygen. Silver, calcium, and phosphorous were homogeneously distributed in the coating. The signal of titanium most probably came from the passivated layer formed before deposition on the NiTi substrate. 

**Figure 3:**
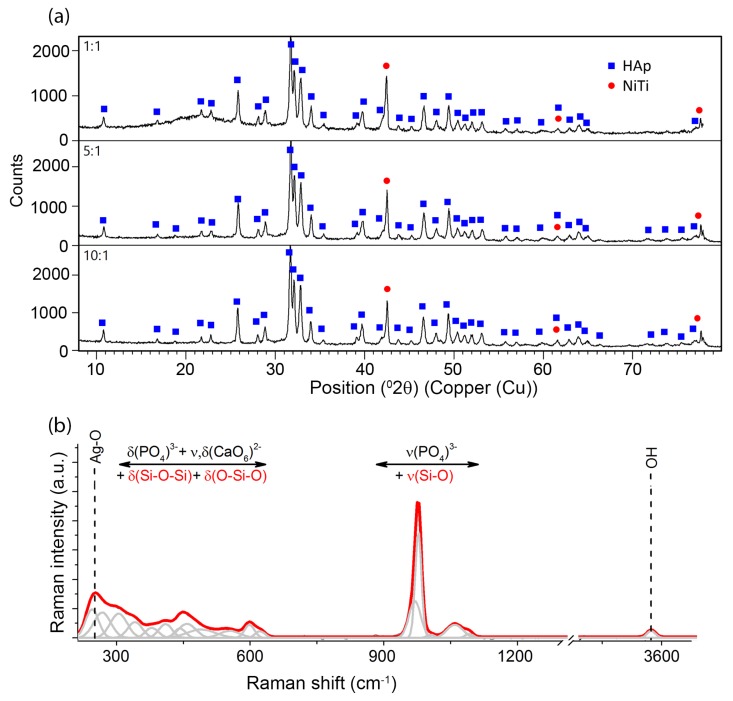
XRD patterns collected for hybrid coatings with different HAp: nSiO_2_/Ag ratios deposited under following parameters: 1:1 50 V/240 s, 5:1 50 V/120 s, and 10:1 40 V/90 s (**a**). Exemplary Raman spectrum of a coating composed of the co-deposited HAp/nSiO_2_/Ag on the NiTi surface. The bands were fitted using Voigt function with the minimum number of components (**b**).

Phase identification that was carried out on the basis of a representative XRD pattern (Figure 3a) revealed the presence of crystalline hydroxyapatite Ca_5_(PO_4_)_3_OH with a hexagonal crystal system (P6_3_/m). The diffraction lines belonging to phase B2 of the NiTi alloy with cubic symmetry (Fd-3m) were also identified. No diffraction lines that belonged to other phases were observed. However, a distinct increase in the background in the range of 14–27 2θ observed for coatings prepared in 1:1 weight ratio and a slight increase in the case of the 5:1 ratio coatings resulted from the existence of the amorphous phase, thus confirming the presence of the silver-silica nanocomposite. The amount of the nSiO_2_/Ag nanocomposite was below the detection limit of the X-ray method for 10:1 coating. Crystalline silver peaks were not observed and the applied deposition parameters did not change the starting materials’ structure.

Unfortunately, there was no possibility to explicitly confirm nSiO_2_/Ag while using XRD. Therefore, another technique has to be applied to examine this component more precisely. One of the techniques providing more information on the structure of nanocomposites is Raman spectroscopy (Figure 3b). The Raman spectra were collected from ten points of different parts of the sample, so as to check the local differentiation of the crystal structure of individual components including the coat-forming material. A great similarity between the Raman spectra allows for their averaging and conducting a global analysis for the entire material. A similar analysis was performed for homogenous coatings that were prepared with a different content ratio of calcium phosphates and silica-silver composites (1:1, 5:1, 10:1). In this approach, the strongest bands that are located between 1000–950 cm^−1^ may be attributed to symmetric stretching vibrations within ν_1_(PO_4_)^3−^ [51,52,53,54,55,56]. Two different tetrahedral surroundings are found in the structure of hydroxyapatite implicate activation of two different modes, which are visible on the Raman spectrum in the form of two bands located at 966 and 955 cm^−1^. Interestingly, a small shift towards lower frequencies in comparison to literature data found for the ideal HAp may result from the insignificant modification of the HAp crystal structure. Some explanations may refer to the presence of a mobile ionic form of Ag in the colloidal suspension, which, due to collisions with the calcium phosphate particles, may lead to the formation of a more disordered HAp system [57]. This effect might intensify due to the local melting of the inorganic materials, especially in the contact zone between nanometer-sized particles. A similar effect was observed in the case of tricalcium phosphate that was deposited with the participation of silver ions or nanoparticles [35]. The remaining bands located in the regions of 1190–1020 and 635–560 cm^−1^ resulted from symmetry-breaking vibrations (ν_3_, ν_2_) within (PO_4_)^3−^ tetrahedrons, as well as might be affected by the presence of silica modes. An interesting situation was observed in the case of low-frequency regions, ranging from 460–400 cm^−1^ (1) and 350–150 cm^−1^ (2). According to the literature, the region (1) is connected to the deformational modes of O-P-O within (PO_4_)^3−^ tetrahedrons. However, the shape and intensity of the fitted line in this region might only be explained by the impact of silica with the stretching and deformation vibration of Si-O-Si and O-Si-O [49]. As a result, the superposition of bands that are attributed to the modes of O-P-O from HAp, Si-O-Si, and O-Si-O of silica enables that reproduction of the real shape observed during the experiment, but makes it impossible to provide a more precise interpretation of the silica component’s nature. The band arrangement of the region (2), similarly to the previous one, is the superposition of the overlapping signal from the vibrations of the entire molecular fragments in Ca(PO_4_) i.e., characteristic for O-Ca-O, O-P-O lattice modes of translational or liberational character, as well as modes that are linked to Ag-O [49]. The low-intensity band that is located at 3573 cm^−1^ is characteristic of vibrations related to hydroxyl groups (-OH^−^) [51,52,53,54,55,56].

### 3.2. Heat Treatment

**Figure 4:**
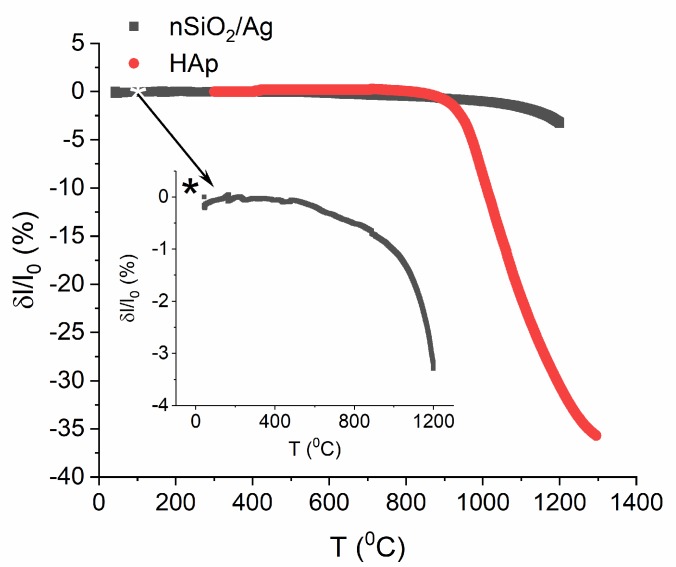
Changes in the powders’ linear dimensions versus temperature for hydroxyapatite powder (red) and nanocomposite nSiO_2_/Ag (black).

Heat treatment was applied to increase the adhesion, density, and bonding strength of electrophoretically deposited composite coatings to the NiTi substrate. It is worth considering the problem of thermal expansion, especially in the context of potential coat-forming materials’ shrinkage, given the high-temperature conditions. Inappropriate thermal conditions may induce internal stresses in the material, which might lead to the cracking and, finally, the delamination of coatings. Therefore, measurements of the initial material’s (HAp, nSiO_2_/Ag) thermal expansion were carried out to determine the conditions of the heat treatment of coatings after the deposition on the NiTi substrate (Figure 4). Hydroxyapatite powder started to shrink at the temperature of 815 °C and then reached a maximum value of 36% at 1300 °C. Shrinkage of the nSiO_2_/Ag nanocomposite began at a lower temperature (ca 490 °C), reaching its maximum value of 3.3% at 1200 °C. The inset in Figure 4 is an enlarged view of the nanocomposite data. Thermal expansion turned to be very low in both cases.

In lower temperatures silica is more sensitive to sintering than hydroxyapatite, and heat treatment should be carried out within a temperature range of 500–800 °C, according to thermal expansion studies. However, too low sintering temperature, such as 500 or 600 °C, might not be sufficient for increasing the adhesion of deposited coatings to the metallic substrate. On the other hand, high sintering temperatures (above 1000 °C) may destroy the NiTi substrate and make it lose its unique features. It seems that the most optimal sintering conditions should be 600–800 °C, although the literature quotes the temperature range of 700–1300 °C for deposited hydroxyapatite coatings [42,44,58,59]. Unfortunately, there are no studies that illustrate the most optimal temperature for the sintering of coatings that are composed of silica-silver material as well as a combination of calcium phosphates and silica-silver. Therefore, the deposited coatings were sintered at 700 °C for 2 h under a protective atmosphere of argon.

### 3.3. Microstructure, Structure and Topography of Coatings after Heat Treatment

**Figure 5:**
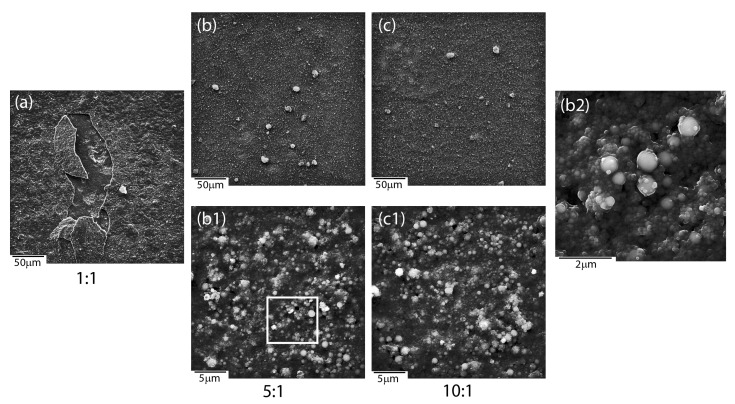
SEM images of coatings with different HAp:nSiO_2_/Ag after heat treatment: 1:1 (**a**), 5:1 (**b**,**b1**,**b2**) and 10:1 (**c**,**c1**).

The microstructure analysis of the initial coatings in relation to the sintered ones, regardless of the mutual weight ratios between ceramic materials (1:1, 5:1, 10:1), revealed the coalescence of ceramics particles, which was particularly well visible at high magnification (Figure 5b2). Tiny material particles were more prone to coalescence than bigger ones. Melted surfaces were observed around bigger particles. A synergic interaction between coat-forming materials, especially silver nanoparticles, might induce the melting of two different materials, such as silica and HAp, which might form a new type of SiO_2_-HAp composite. Unfortunately, it is impossible to provide a clear explanation merely on the basis of microstructure analysis. Another problem that was related to 1:1 ratio coatings after the heat treatment was the appearance of cracks. It turned out that these layers are prone to delamination and chipping, which practically prevents their potential use when developing a functionalized implant material (Figure 5a). Therefore, the 1:1 ratio coating was not considered during further analysis. The other layers turned to be highly continuous and free of cracks in the entire coating (Figure 5b,c). 

**Figure 6:**
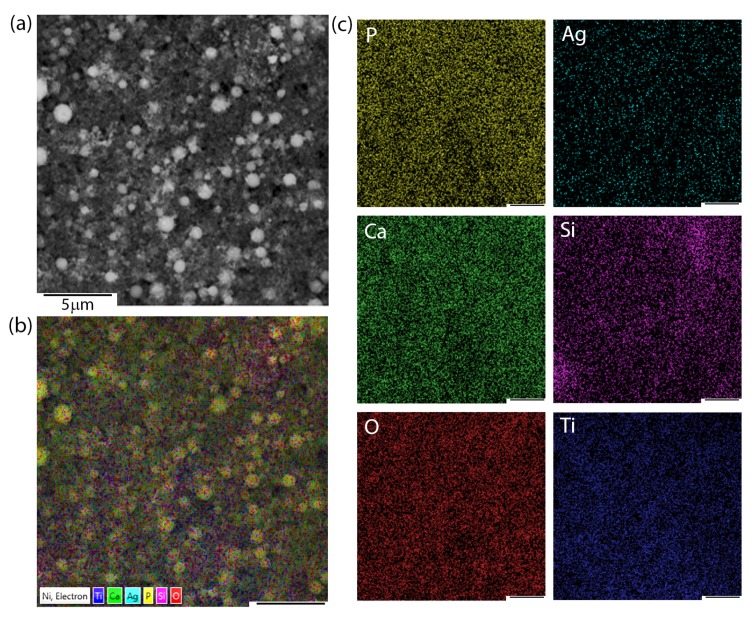
SEM image of the 5:1 coating (**a**), multi-layer elemental distribution (**b**) and chemical composition imaging of elements (**c**) after heat treatment.

Another problem that was linked to the heat treatment turned out to be the risk of metal evaporation from the coat-forming composite (e.g., silver). The problem of silver evaporation might lead to the deterioration of antimicrobial properties, which belong to the most important potential features of hybrid coatings. According to the literature data, silver (ionic or as nanoparticles) in the similar nSiO_2_/Ag material only evaporates after exceeding the temperature of 1080 °C, whereas it should remain unchanged at lower temperatures [60]. The SEM + EDS data indicate that silver content, both before and after sintering, was similar, reaching (1.5 ± 0.3 wt.%) and (0.7 ± 0.2 wt.%), for 5:1 and 10:1 ratio coatings, respectively. A relatively low silver content should have no, or merely a very low negative impact on the proliferation of fibroblasts according to the previous reports, allowing for the material to preserve its antimicrobial properties [35]. The elemental distribution analysis that was performed for a sintered NiTi alloy functionalized with a coating composed of HAp and nSiO_2_/Ag revealed homogenous silver distribution in the coat-forming material (Figure 6). The data refers to exemplary 5:1 ratio coating, while similar chemical images were obtained for the 10:1 layer. Interestingly, silver had no tendency to agglomerate and remained uniformly dispersed in the composite hydroxyapatite-silica layer. Other elements, such as calcium, phosphorus, as well as silicon and oxygen, were also homogeneously distributed throughout the coating. These data indicate that HAp and silica were homogenously distributed in the layer and in relation to each other. Titanium from the intermediate titanium oxide layer was also homogeneously distributed in the coating.

**Figure 7:**
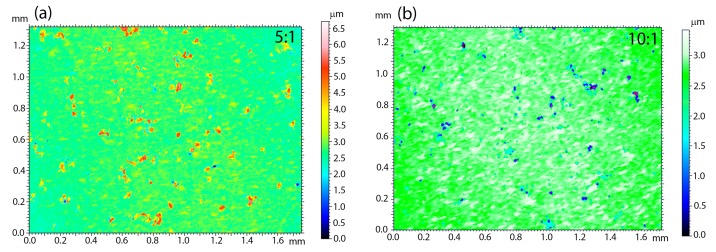
3D topography of surface layers 10:1 (**a**) and 5:1 (**b**).

An important issue related to the creation of a new type of coatings is the analysis of surface parameters, such as roughness. These parameters determine surface development, mainly in the context of osseointegration studies. The higher the roughness value is, the better is the ability of the cell to proliferate and the shorter healing process, according to the literature [61]. It is therefore worth looking at the quality and roughness of the coatings prepared as a combination of HAp and a silica-silver nanocomposite. It is known that these features are strictly associated with the deposition parameters and they may be subjected to modification after sintering. In view of the above and the applied method of making coatings that functionalize the NiTi alloy, only the topography of the sintered material was considered. Moreover, SEM investigations revealed the poor quality of 1:1 coatings; therefore, the 3D surface topography visualizations were performed for the 5:1 (Figure 7a) and 10:1 coatings (Figure 7b). Interestingly, the coatings’ roughness decreases with a lower nanocomposite content in the colloidal suspension. The average roughness (*S*_a_) that was estimated for 5:1 and 10:1 reached 0.20(3) μm and 0.14(2) μm, respectively. For biomedical applications, a better solution seems to be the hybrid coating that was obtained in the process of electrophoretic deposition with a slightly higher weight content of silica-silver nanocomposite in relation to hydroxyapatite.

**Figure 8:**
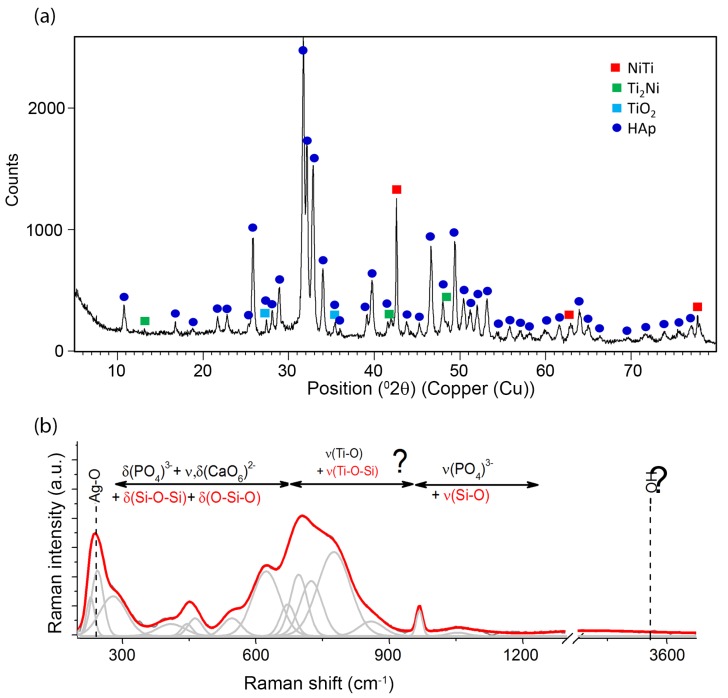
XRD pattern collected for coating 5:1 (50 V, 120 s) after heat treatment (**a**) and exemplary Raman spectrum. The bands were fitted using Voigt function with the minimum number of components (**b**).

Structural investigation that was based on X-ray measurements revealed the presence of main diffraction lines belonging to crystalline hydroxyapatite (Figure 8a). Similar diffraction patterns were observed for all coatings with different weight ratios between ceramics, which indicated that the applied heat treatment conditions did not result in the decomposition of the calcium phosphate material. However, the applied heat treatment temperature influenced the crystallization of rutile with a tetragonal lattice (P4_2_/mnm) and caused partial decomposition of the NiTi alloy to the equilibrium Ti_2_Ni phase with a cubic lattice (Fd-3m). Similar observations were previously quoted in the subject literature [10,11]. We can also speculate that an increase in sintering temperature might result in the crystallization of silica in the amount visible in XRD investigations, according to ref. [60]. Similarly to X-ray patterns after electrophoretic deposition (Figure 3a), there was no clearly visible evidence regarding the presence of silica and its impact on the coat-forming material. The same problem concerns the determination of silver. However, the lack of diffraction lines from silver might be correlated with its very low content and/or nanometer size, according to SEM + EDS studies.

The Raman spectroscopy approach has provided more information on the structure of the coating that was subjected to heat treatment. Similarly to the initial coatings, ten Raman spectra were collected from different parts of the coating. Their high similarity allowed for their averaging and analyzing as a representative of the whole material. The same spectra were obtained for both coatings that were prepared in 5:1 and 10:1 mutual ratio between HAp and nSiO_2_/Ag (Figure 8b). The point Raman analysis revealed some interesting structural changes after heat treatment, which were completely unexpected, difficult to explain, and invisible in X-ray data. One low-intensity band that centered at around 967 cm^−1^ is associated with the symmetric stretching of ν_1_ vibration of P-O within the (PO_4_)^3−^ tetrahedra and it is a typical marker band that characterizes hydroxyapatite. The position of this line is very close to the band position of hydroxyapatite in the sample that has not been subjected to heat treatment. Unfortunately, the interpretation of other marker bands of HAp is difficult due to the presence of other strong-intensity bands. As a result, it is not possible to find the ν_4_, ν_2_, ν_3_ modes, which probably overlap other bands. The low intensity of the main HAp band might correspond to the lack of a clearly visible signal of hydroxyl groups, which, in typical calcium phosphates, is represented by a low-intensity band (Figure 3b) [57]. A different situation is observed in the case of silica and silver. The strong-intensity band that is centered around 240 cm^−1^ is attributed to the Ag-O modes, whereas a significant increase in band intensity in the sintered sample related to the fact that the material was not subjected to heat treatment indicates silver oxidation. At the same time, a relatively low-intensity Ag-O band in the initial material might point to the presence of ionic, metallic, and/or oxidized silver. One of the possible explanations of the silver oxidation might be its binding to silica or calcium phosphate particles. The area around the HAp particles observed in SEM images (Figure 5b2) might be due to the surface merging of silver with calcium phosphate as a result of HAp surface melting, according to this assumption. Alternatively, a similar hypothesis might be considered with silica carrier, especially that typical silica bands found between 460–400 cm^−1^ increase their intensity after coating sintering (Figure 8b). The other bands that are characterized by very strong intensity are extremely difficult to interpret and there are no literature data highlighting this problem. However, two bands that are centered at around 600 and 450 cm^−1^ may be associated with the stretching vibrations of Ti-O, which are typically characteristic of the rutile structure and they correspond to XRD data. Other strong-intensity bands of a relatively high full width at half maximum values may suggest the formation of strongly disordered or even amorphous composite materials. However, to provide a more precise explanation of the nature of such a phase from the structural point of view, it is necessary to correlate the Raman data with SEM + EDS findings (Figure 6). The main elements (Ca, P, Si, O, Ti) are homogeneously distributed in the coating, indicating that the band arrangement visible on the Raman spectrum might correspond to the formation of titanium-silica-calcium phosphate or titanium-silica disordered structure, according to the chemical composition analysis. However, the first system seems to be relatively less likely due to the thermal stability of calcium phosphate. Consequently, a possible interpretation of the band arrangement ought to be sought in the formation of the TiO_2_-SiO_2_ system as a result of the synergistic interaction between silver (ionic, metallic, or oxidized form), temperature, and the amorphous nature of titanium and silica. The temperature and the phase diagram of crystalline TiO_2_ and SiO_2_ indicate that the eutectic point of stable TiO_2_-SiO_2_ phase formation appeared at temperatures of ca 1400 °C, or even higher, but at a low molar content of silica in the system [62]. Of course, this temperature is too high in relation to that applied for the HAp/nSiO_2_/Ag coating that was deposited on the NiTi alloy, but it could be a starting point for further discussion on the mechanism of the coating formation after sintering. Amorphous materials, such as silica (coat-forming material) and titanium oxide (passivated NiTi layer), due to the disordered structure, may be much more thermally unstable than their crystalline counterparts, according to some hypotheses. Moreover, silver in the system might play the role of a catalyst lowering the temperature of the system formation. There is a likelihood that the applied heat treatment conditions forced the formation of the disordered phase system at a much lower temperature than crystalline systems that formed at considerably higher temperatures. However, this is only a hypothesis, which ought to be proven using another technique. 

### 3.4. Bonding Strength and Ability of Layers to Deform

The bonding strength between the hybrid coatings 5:1 and 10:1 that were obtained after sintering and the passivated NiTi substrate, measured by a shear strength test, was (13.8 ± 1.8) MPa and (14.9 ± 1.2) MPa, respectively. It accounts for approximately 37% (for the 5:1 coating) and ca 40% (for the 10:1 coating) of the shear strength of the cortical bone (34 MPa) [63,64]. Similar bonding strength values were obtained for layers of undoped hydroxyapatite that was electrophoretically deposited on the NiTi alloy and sintered at 800 °C [10]. It is known that applying a higher heat treatment temperature results in an increase of ceramic layers’ adhesion to the metallic substrate. A similar result that was obtained for HAp/nSiO_2_/Ag hybrid layers sintered at 700 °C might be due to the formation of a completely new phase (Figure 8b), which increases the adhesion parameters. The formation of a strongly disordered phase was previously reported for electrophoretically deposited β-TCP + Ag/SiO_2_ coatings [35].

**Figure 9:**
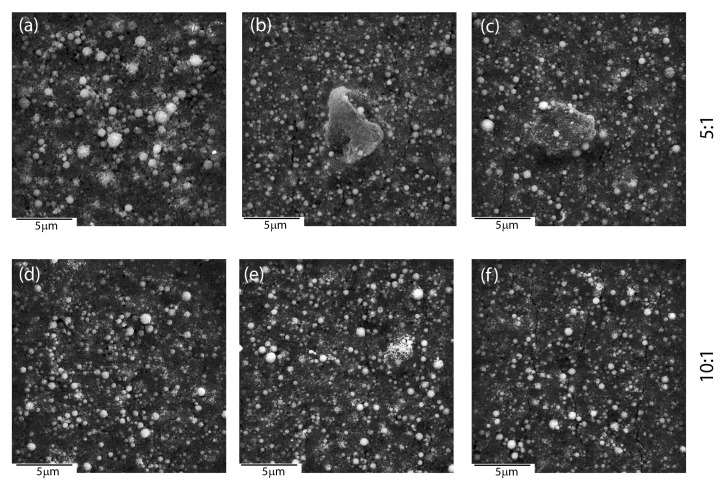
SEM image of the surface of the 5:1 (**a**–**c**) and 10:1 (**d**–**f**) HAp/nSiO_2_/Ag layer sintered at 700 °C/2 h after deformation.

It is wort nothing that the layers that formed on shape memory alloys should also be capable of deforming. Tensile stress is crucial for ceramic coatings. Therefore, the samples of both coatings (10:1 and 5:1) were subjected to deformation until the plastic deformation of the NiTi substrate was achieved (Figure 9). The first microcracks of the 10:1 layer were observed at ε = 2.02% (Figure 9d). Increasing the deformation resulted in an increase in the length of the cracks and, at ε = 3.23% (Figure 9e), the formation of their fine system on the entire surface of the sample was visible. At the maximum applied deformation ε = 3.57% (Figure 9f), the cracks increased in size, but no layer delamination of the NiTi substrate was observed. In the case of the 5:1 layer with higher roughness, in the process of deformation, cracks should be expected to appear in the area of larger agglomerates, as it is in this location where the biggest stresses are generated. However, the first break in coating continuity was observed in the areas between agglomerates, with deformation that was similar to that of the 10:1 layer ε = 1.96% (Figure 9a). Crack propagation in the layer and an increase in the cracks’ size were observed as the deformation increased (Figure 9b). However, even with the largest deformation ε = 3.47% (Figure 9c), there was no agglomerates spalling or layer delamination. As reported in [10], the non-doped hydroxyapatite layers displayed similar behavior with a similar range of deformations. The deformation behavior does not depend on the composition and roughness of the coating.

### 3.5. In Vitro Corrosion Resistance Tests

One of the challenges faced in the production of medical implants is the use of short-term in vitro tests for assessing the long-term in vivo corrosion behavior of implants. The methodology of the in vitro electrochemical testing of the investigated NiTi shape memory alloy with HAp/nSiO_2_/Ag hybrid coatings involves measurements of electrical quantities during free corrosion (for open circuit), in potentiodynamic and impedance tests, which allow for faster assessment of material resistance to any corrosive phenomena, mainly pitting corrosion in the living organism environment.

**Figure 10:**
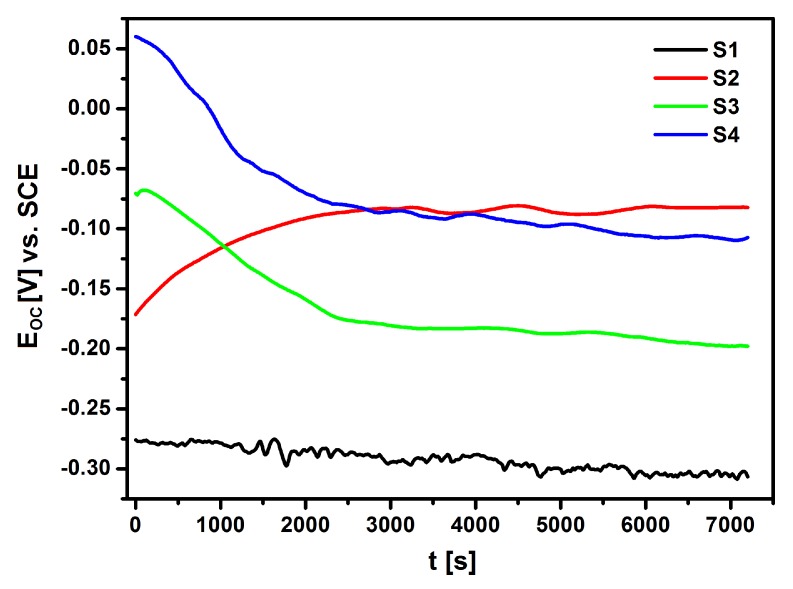
Open circuit potential for the NiTi alloy before and after surface modification in Ringer’s solution at 37 °C, where: S1—NiTi alloy after mechanical polishing, S2—NiTi alloy after passivation in a steam autoclave, S3—NiTi alloy after sterilization with a sintered HAp/nSiO_2_/Ag coating (10:1, 40 V/90 s) and S4—NiTi alloy after sterilization with a sintered HAp/nSiO_2_/Ag coating (5:1, 50 V/120 s).

In the measurements that were conducted by the open circuit potential method, the difference was measured and the rate of stabilization of the potential value between the tested electrode and the reference electrode was recorded without applying an external current source to the system. The stabilized *E*_OC_ value was considered to be an approximate value of the corrosion potential. The *E*_OC_ method made it easy to assess the protective properties of the passive layers that formed on the surface of the NiTi alloy and the corrosion resistance of the hybrid coatings applied. Figure 10 shows the results that were obtained in the form of *E*_OC_ dependence as a function of time for NiTi electrodes before and after surface modification in Ringer’s solution.

The *E*_OC_ of the electrodes that were immersed in the electrolyte changed significantly over time. The ionic-electron equilibrium on the electrode|electrolyte interface was determined approximately 120 min. after the immersion of the electrodes in the electrolyte. Based on the obtained results, it can be concluded that the lowest corrosion resistance was demonstrated by the S1 electrode made of NiTi alloy after mechanical polishing (*E*_OC_ = −0.306 V). The application of NiTi electrode surface modification processes in the form of sterilization and deposition of the HAp/nSiO_2_/Ag hybrid coating after subsequent sintering significantly improved the corrosion resistance of the NiTi alloy. The *E*_OC_ of so modified NiTi electrodes was −0.082 V for the NiTi alloy after passivation in a steam autoclave (S2), −0.198 V for the NiTi alloy after sterilization with a sintered HAp/nSiO_2_/Ag coating (10:1, 40 V/90 s) (S3) and −0.107 V for the NiTi alloy after sterilization with a sintered HAp/nSiO_2_/Ag coating (5:1, 50 V/120 s) (S4). The S2 electrode with an ultra-thin passive oxide film exhibited the strongest barrier properties.

**Figure 11:**
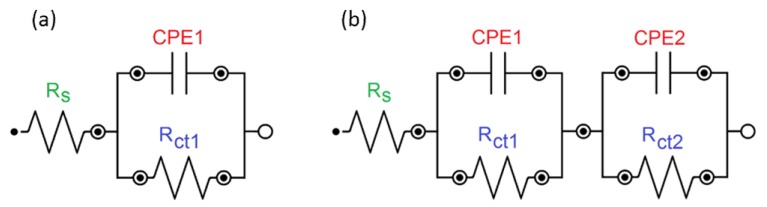
Equivalent electrical circuit for the NiTi|oxide layer|Ringer solution system (**a**) and the NiTi|oxide layer|composite HAp/nSiO_2_/Ag coating|Ringer solution system (**b**) in the pitting corrosion process.

The impedance tests were carried out in order to determine the mechanism and kinetics of corrosive processes occurring on the electrode|electrolyte interface during the exposure of the received materials in the Ringer’s solution. The electrode potential was stabilized until a constant *E*_OC_ value was obtained due to the fact that the EIS method is only applicable to electrochemical systems that behave linearly and are in steady-state. For approximation of experimental impedance data that were obtained in the case of S1 and S2 electrodes, the simplest electrical equivalent circuit in the form of the Randles circuit was used [46,47,48]. A solution resistance (*R*_s_) in series with a parallel connection of a double layer capacitance (*C*_dl_) and a charge transfer resistance (*R*_ct_) was used to model the AC impedance data. When considering that measured capacitance usually deviates from the pure capacitance due to the electrode surface roughness, the *C*_dl_ was expressed in terms of the constant phase element (CPE), the impedance of which is given by:

(1) $${\hat{Z}}_{\mathrm{CPE}}=\frac{1}{T{\left(j\omega \right)}^{\phi }}$$

where: *T* is the capacitance parameter given in F cm^−2^ s*^ϕ^*^−1^ dependent on the electrode potential, and *ϕ* relates to the angle of rotation of purely capacitive line on the complex plane plot plots: *α* = 90° (1−*ϕ*). CPE is associated with a leaking capacitor with non-zero real and imaginary components. For *ϕ* = 1, purely capacitive behavior is obtained and *T* = *C*_dl_. In Equation (1), the *T* parameter represents pure capacitance for *ϕ* = 1, infinite Warburg impedance for *ϕ* = 0.5, pure resistance for *ϕ* = 0, and pure inductance for *ϕ* = −1. Figure 11a shows the described model that represents the system of the NiTi|oxide layer|Ringer solution. It produces one semicircle on the complex plane plot (*R*_s_, CPE-*T*_1_, CPE-*ϕ*_1_, *R*_ct1_). Such a model is well-known in the testing of corrosion resistance of NiTi alloy in a simulated human body solution [46,47,48].

In the case of S3 and S4 electrodes, a more complex model that considers the inner compact oxide layer and the outer porous layer in contact with the electrolyte was proposed to study the interfacial properties of the NiTi|oxide layer|composite HAp/nSiO_2_/Ag coating|Ringer solution system. This model consists of two Randles circuits in series and it produces two semicircles on the complex plane plot (Figure 11b). The high frequency (HF) semicircle is related to the composite HAp/nSiO_2_/Ag coating|Ringer solution interface and it is described by *R*_s_, CPE-*T*_1_, CPE-*ϕ*_1_, and *R*_ct1_ parameters, whereas the low frequency (LF) semicircle is associated with the oxide layer|Ringer solution interface and it is described by CPE-*T*_2_, CPE-*ϕ*_2_, and *R*_ct2_ parameters.

**Figure 12:**
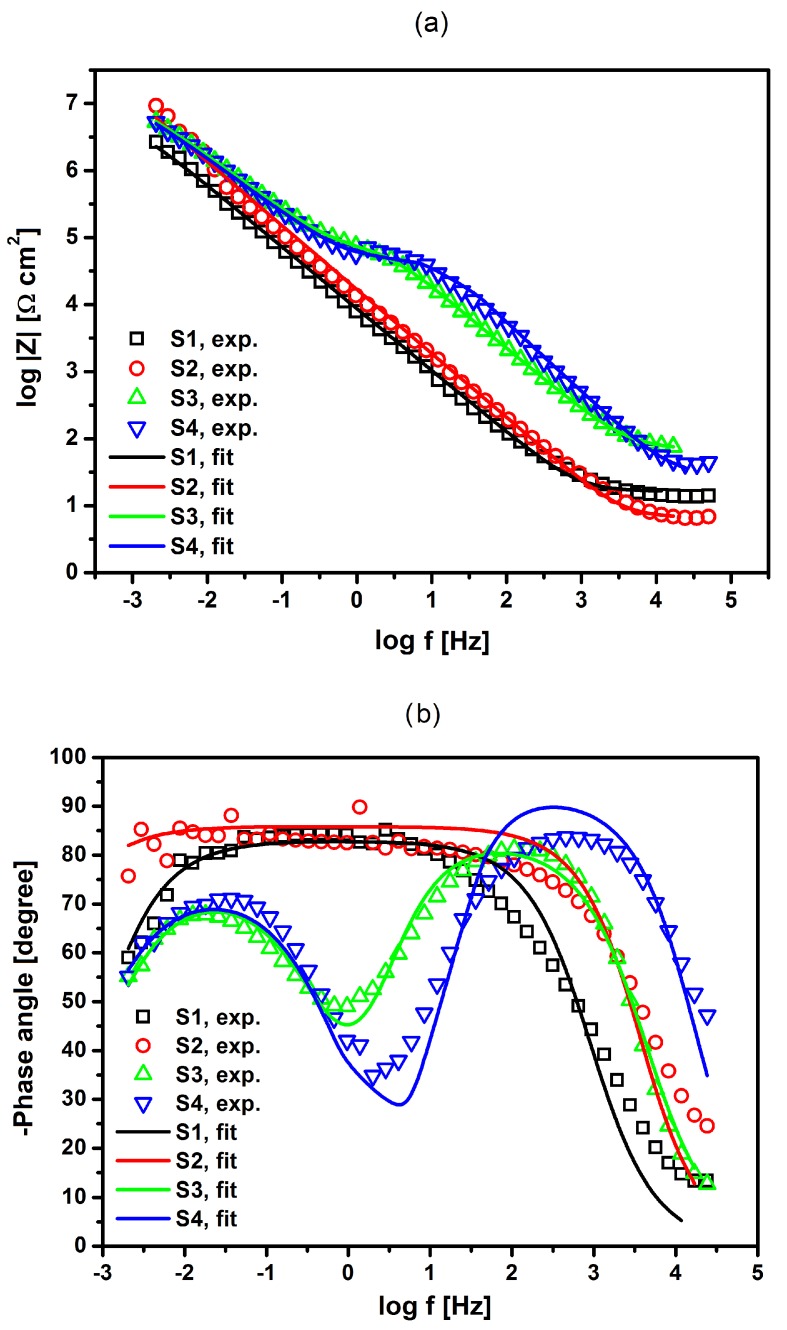
Bode plots log |Z| vs. log *f* (**a**) and Bode plots *φ* vs. log *f* (**b**) for the NiTi alloy before and after surface modification in Ringer’s solution at 37 °C. Experimental AC impedance data are represented by symbols; fitting results obtained using CNLS method and models shown in Figure 11 are shown as continuous lines. Legend: S1—NiTi alloy after mechanical polishing, S2—NiTi alloy after passivation in a steam autoclave, S3—NiTi alloy after sterilization with a sintered HAp/nSiO_2_/Ag coating (10:1, 40 V/90 s) and S4—NiTi alloy after sterilization with sintered HAp/nSiO_2_/Ag coating (5:1, 50 V/120 s).

Figure 12a,b show Bode plots showing the dependence of the impedance module logarithm and phase angle (*φ*) as a function of the frequency logarithm, respectively. One can observe a very good fit of the experimental impedance data and theoretical models shown in Figure 11 for the NiTi alloy before and after surface modification in Ringer’s solution. In the case of S1 and S2 electrodes that are covered with an oxide film, only one semicircle was observed on the complex plane plots with one time constant. For S3 and S4 electrodes with a composite HAp/nSiO_2_/Ag coating, two semicircles were visible on the complex plane plots with two-time constants, where the diameter of the LF semicircle was higher. The value of the slope in the form of the impedance module in the medium frequency range was about −1, according to theoretical predictions. The measured high values of |*Z*|_f__→0_ and *φ* close to −90° confirm the capacitive behavior of the material that was characterized by high corrosion resistance. The obtained impedance values are in good compliance with the experimental impedance that was determined for passivated metallic materials in solutions containing aggressive Cl^−^ ions, which cause pitting corrosion [46,47,48]. One can also observe an increase in the value of log|*Z*| at the lowest frequency of 2 mHz for S2, S3, and S4 electrodes in comparison with that determined for S1 electrode, which indicates that the methods that are used to modify the surface of the NiTi electrode increase its corrosion resistance in Ringer’s solution (Figure 12). The highest corrosion resistance can be confirmed for the NiTi alloy after autoclaving (S2).

Table 2 presents the summary of the parameters that were obtained as a result of approximating the experimental EIS data for the NiTi alloy before and after surface modification using the proposed models of equivalent electrical circuits in the pitting corrosion process.

The error of the particular parameter determination was below 25%. It can be clearly seen that there are evident changes between the characteristics of the oxide film and the HAp/nSiO_2_/Ag composite coating. The *R*_ct1_ parameter that corresponds to the oxide layer|electrolyte interface is characterized by the highest value of 5.9 × 10^7^ Ω cm^2^ for S2 electrode and it indicates its strongest protective properties. The obtained result is consistent with the *E*_OC_ measurements (Figure 10). The *R*_ct1_ values for S3 and S4 electrodes related to the HAp/nSiO_2_/Ag composite coating|electrolyte interface are lower by 10^4^ Ω cm^2^. However, it should be noted that the sub-surface oxide layer is still present and it retains its effective barrier properties (*R*_ct2_). A significant deviation of CPE-*ϕ*_2_ from 1 for S3 and S4 electrodes indicates a change in the CPE2 behavior, which can be associated with the presence of a porous HAp/nSiO_2_/Ag composite coating having different physical, chemical, and geometrical factors when compared to the smooth oxide barrier layer that is represented by CPE-*ϕ*_1_. The values of CPE-*ϕ*_2_ of 0.831 to 0.825 were obtained for S3 and S4 electrode, respectively. The presence of Ni was not detected on the surface of the tested electrodes after the corrosion tests.

**Figure 13:**
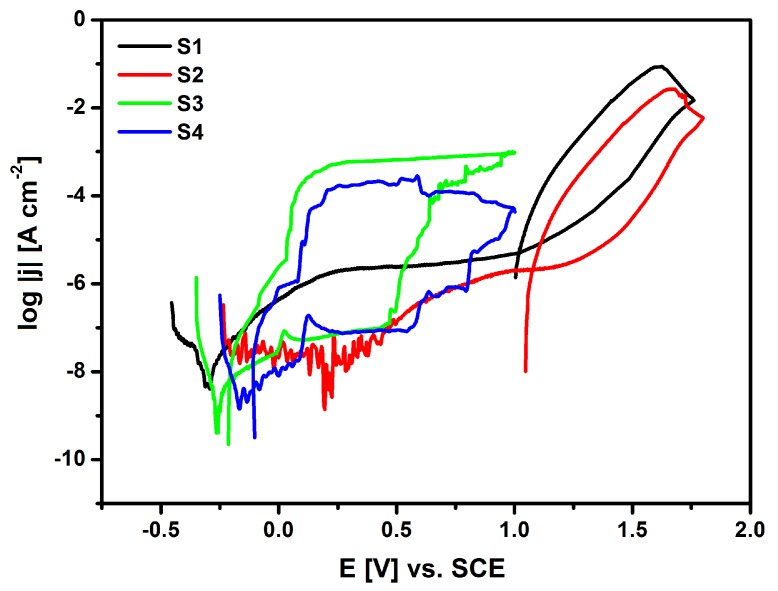
Anodic potentiodynamic curves *j = f(E)* on a semi-logarithmic scale for the NiTi alloy before and after surface modification in Ringer’s solution at 37 °C, where: S1—NiTi alloy after mechanical polishing, S2—NiTi alloy after passivation in a steam autoclave, S3—NiTi alloy after sterilization with a sintered HAp/nSiO_2_/Ag coating (10:1, 40 V/90 s), and S4—NiTi alloy after sterilization with a sintered HAp/nSiO_2_/Ag coating (5:1, 50 V/120 s).

The susceptibility of the tested materials to pitting corrosion in the biological environment was determined on the basis of recorded cyclic anode polarization curves that were obtained by the potentiodynamic method The measurement was based on a continuous change of the electrode potential at a sweep rate of 1 mV s^−1^ with simultaneous recording of the current flowing through the interface of electrode|Ringer solution. The anodic potentiodynamic curves were prepared on a semi-logarithmic scale log *j = f(E)* and are shown in Figure 13. Table 3 shows the key electrochemical parameters for the NiTi alloy before and after surface modification determined on the basis of potentiodynamic measurements.

It can be stated that after the applied surface modifications of the NiTi alloy, a shift of the minimum value corresponding to *E*_cor_ towards the anode potentials is observed in Figure 13 for S2, S3 and S4 as compared to *E*_cor_ on log *j = f(E)* curve for the NiTi electrode after mechanical polishing (S1), based on the obtained potentiodynamic characteristics in a wide range of potentials. Corrosion potential is widely recognized as a parameter that allows for a preliminary assessment of the corrosive properties of metals and alloys. This parameter makes it possible to predict when destructive processes will begin in the tested material that is exposed to a corrosive environment. The corrosion current density (*j*_cor_) value is directly proportional to the rate of the electrochemical corrosion process. However, it cannot be used as a kinetic parameter to compare the corrosion resistance of the studied materials. The NiTi alloy has the ability to self-passivate in the presence of an oxygen carrier, so the corrosion potential of the NiTi electrode after the mechanical polishing process (S1) is in the passive region of the obtained potentiodynamic characteristics. The corrosion resistance of the tested electrodes mainly depends on the structure and thickness of the passive layer. The oxide layer in the form of a nanometric TiO_2_ layer is formed on the NiTi alloy surface as a result of its contact with air. This reaction occurs spontaneously. The highest value of *E*_cor_ = 0.195 V was determined for the S2 electrode after forced passivation in an autoclave.

The deterioration of the tested materials’ corrosion resistance is associated with chloride ions that are present in the solution responsible for pitting. Pitting is caused by the interaction of halide ions, such as Cl^−^, on the surface of the passive layer, which have the ability to destroy it locally. Chloride ions accumulate on the surface of the passive layer and penetrate deep into the material where it is the weakest, e.g., at grain boundaries or in the place of mechanical damage. The Okamoto model describes the phenomenon of pitting initiation and propagation. It was discussed in our earlier work [48]. It is assumed that the water molecules on the metal surface are replaced with chloride ions. By bonding with metal, these ions hinder the incorporation of metal ions into the passive layer, making it easier for them to go into the solution and, thus, inhibit the repassivation phenomenon. A corrosive cell forms at the pitting site. The surrounding surface is the cathode, where oxygen reduction occurs, whereas the inside of the pitting is the anode, and this is where the digestion of metal takes place, as a result of which the pitting grows.

In the range of potentials from *E*_cor_ up to the breakdown potential, the tested electrodes are in the passive area and they have various values of anode current densities, which result from the phase composition, chemical composition, and the thickness of the layers. For all of the recorded anode polarization curves at *E*_b_, an increase in the density of the measured current is observed, which is due to the initiation of pitting corrosion (Figure 13). Table 3 presents the determined *E*_b_ values, together with the corresponding values of the breakdown current density (*j*_b_) for NiTi electrodes before and after surface modification. It is worth emphasizing that the highest value of the breakdown potential in Ringer’s solution containing aggressive chloride ions was determined for the NiTi electrode after autoclaving (*E*_b_ = 1.675 V), which confirms its highest corrosion protection among the tested electrode materials. Above the *E*_b_ value, a rapid increase in the current density value can be observed, which is caused by an effective oxygen release and anodic dissolution of the electrodes. The hysteresis loops that are presented in Figure 13 for S1, S2, S3, and S4 electrodes occurred at the reverse polarization of the width that was dependent on the type of the layer coating the NiTi electrode surface. Their appearance indicates the development of formed pits. The potential at which the return curves of log *j = f(E)* intersect the primary polarization characteristics is treated as a protection potential (*E*_p_). Below the E_p_, the existing pits are repassivated and new pits are not formed. Table 3 shows the key parameters, such as *E*_b_ and *E*_p_ for the studied electrodes in Ringer’s solution. The large width of the hysteresis loop (*E*_b_ *− E*_p_) in Figure 13 for S3 and S4 electrodes indicates that the HAp/nSiO_2_/Ag composite coatings exhibit much higher susceptibility to pitting corrosion in Ringer’s solution when compared to the NiTi electrode after mechanical polishing (S1) and autoclaving (S2). The lowest key electrochemical parameters were observed for the NiTi alloy after sterilization with the HAp/nSiO_2_/Ag coating (5:1, 50 V/120 s) that was sintered at 700 °C in argon, probably due to the fact that it has the most developed surface among all of the tested electrodes (Table 3).

## 4. Conclusions

The paper presents first data illustrating the formation of hybrid ceramic coatings consisting of hydroxyapatite (HAp) and silicon dioxide/silver (nSiO_2_/Ag), with different ratios (1:1, 5:1, and 10:1) electrophoretically deposited on the surface of a passivated NiTi shape memory alloy. Deposition parameters allowing for the formation of a homogeneous, crack-free layer covering the entire surface were selected. The applied heat treatment in protective atmosphere (700 °C/2 h) enabled obtaining crack-free coatings only in the case of 5:1 and 10:1 ratios. Spalling and cracks were observed after sintering in the case of 1:1 ratio coating. X-ray measurements revealed that the sintering process resulted in the partial decomposition of the NiTi parent phase and the formation of the equilibrium one (Ti_2_Ni). As a result of heat treatment, the crystallization of titanium oxide and structural changes in the deposited materials were observed. Raman analysis revealed the oxidation of silver as well as the formation of an atypical strongly disordered system, which, in combination with SEM + EDS data, indicates the formation of the TiO_2_-SiO_2_ system. The 5:1 and 10:1 coating was characterized by good adhesion to the NiTi substrate and the ability to deform up to ca ε = 3.5%.

The detailed mechanism and kinetics of the pitting corrosion of the NiTi electrode with and without HAp/nSiO_2_/Ag hybrid coatings were determined in Ringer’s solution, while using EIS data analyzed by the equivalent electrical circuit method, taking the physical meaning of individual elements of the circuit used into account. The experimental and the theoretical data were found to be highly consistent. The AC impedance measurements confirmed the capacitive behavior of the material with high corrosion resistance. The fastest kinetics and the highest susceptibility to pitting corrosion were noted for the NiTi electrode that was coated with HAp/nSiO_2_/Ag hybrid coatings formed at 50 V for 120 s (5:1) and sintered at 700 °C due to high surface porosity.

## Figures and Tables

**Table 1:** Chemical composition of Ringer’s solution.

Component	Concentration (g dm^−3^)
NaCl	8.60
KCl	0.30
CaCl_2_	0.48

**Table 2:** Parameters values obtained using the equivalent circuit models shown in Figure 11 to approximate the experimental electrochemical impedance spectroscopy (EIS) data for the NiTi alloy before and after surface modification in Ringer’s solution at 37 °C (see Figure 12a,b).

No.	CPE-*T*_1_ (F cm^−2^ s*^ϕ^*^−1^)	CPE-*ϕ*_1_	*R*ct_1_ (Ω cm^2^)	CPE-*T*_2_ (F cm^−2^ s*^ϕ^*^−1^)	CPE-*ϕ*_2_	*R*_ct2_ (Ω cm^2^)
S1	3.1 × 10^−5^ ± 7.0 × 10^−7^	0.920 ± 0.005	4.0 × 10^6^ ± 5.3 × 10^5^	-	-	-
S2	1.5 × 10^−5^ ± 2.9 × 10^−7^	0.954 ± 0.003	5.9 × 10^7^ ± 5.9 × 10^5^	-	-	-
S3	1.9 × 10^−6^ ± 9.1 × 10^−8^	0.975 ± 0.011	3.3 × 10^4^ ± 1.7 × 10^3^	8.7 × 10^−6^ ± 1.7 × 10^−7^	0.831 ± 0.007	9.6 × 10^6^ ± 5.6 × 10^5^
S4	3.2 × 10^−7^ ± 4.0 × 10^−8^	1.072 ± 0.019	2.6 × 10^4^ ± 1.9 × 10^3^	9.5 × 10^−6^ ± 3.9 × 10^−7^	0.825 ± 0.015	1.1 × 10^7^ ± 1.9 × 10^6^

**Table 3:** Key electrochemical parameters for the NiTi alloy before and after surface modification after potentiodynamic measurements in Ringer’s solution at 37 °C presented in Figure 13.

No.	*E*_cor_ (V)	*j*_cor_ (A cm^−2^)	*E*_p_ (V)	*j*_p_ (A cm^−2^)	*E*_b_ (V)	*j*_b_ (A cm^−2^)
S1	−0.292	4.1 × 10^−9^	1.020	5.5 × 10^−6^	1.620	8.7 × 10^−2^
S2	0.195	1.4 × 10^−9^	1.078	2.2 × 10^−6^	1.675	2.7 × 10^−2^
S3	−0.265	4.1 × 10^−10^	−0.207	6.5 × 10^−9^	0.475	6.5 × 10^−4^
S4	−0.171	1.6 × 10^−9^	−0.105	4.5 × 10^−9^	0.587	2.8 × 10^−4^
